# Does Aging Identity Moderate the Impact of Experiences with Familial Ageism on Well-Being?

**DOI:** 10.1093/geroni/igaa057.3221

**Published:** 2020-12-16

**Authors:** Emily Kinkade

**Affiliations:** North Dakota State University, Fargo, North Dakota, United States

## Abstract

This study begins to investigate the effects of ageism in the family context. The current literature has documented the negative impacts that negative stereotypes and negative perceptions of aging have on older adults’ health, mortality, and well-being (Levy, 1996; Levy, 2003). However, the majority of extant research on ageism focuses on age discrimination in the workplace and in healthcare despite the majority of peoples’ time being spent in the family context. Therefore examining experiences of ageism sourced from family members merits study. Walker, Bisconti and Kinkade (in preparation) found evidence that the experience of ageism within the family context varies from the workplace context. Past research has demonstrated that older adults who identify as being older and adapt to the changes that arise with aging are associated with higher levels of self-esteem (Whitbourne, Sneed, & Skultety, 2002; Weinberger & Whitbourne, 2010). It is hypothesized that age identification will serve as a moderator for the relationship between familial ageism and well-being. Participants completed a set of questionnaires measuring experiences with familial ageism, depression, self-esteem, and ego strength. Experiences of familial ageism correlated with the well-being outcome variables in the predicted direction. Age identity moderated the relationship between familial ageism and depression and ego strength, such that participants who identified as being younger or identified as their age reported lower depression scores and higher ego strength scores. These findings suggest that age identity may serve as a buffer against the negative impacts that experiences of familial ageism has on well-being.

